# Supplementary material for: Tangent space functional reconfigurations in individuals at risk for alcohol use disorder
Source: ArXiv. 2024 May 24:arXiv:2405.15905v1. Preprint. [Version 1] (PMC11142326)
Supplement: Supplementary file 1 [file NIHPP2405.15905v1-supplement-1.pdf]

Supplementary Material

Table S1. Loadings of recent drinking behavior principal components

	PC 1	PC 2
Loadings		
AUDIT	0.90	0.03
Total drinking days	0.68	-0.71
Drinks per week	0.97	-0.02
Drinks per drinking day	0.63	0.75
Eigenvalues	2.63	1.08
Explained Variance	64%	26%

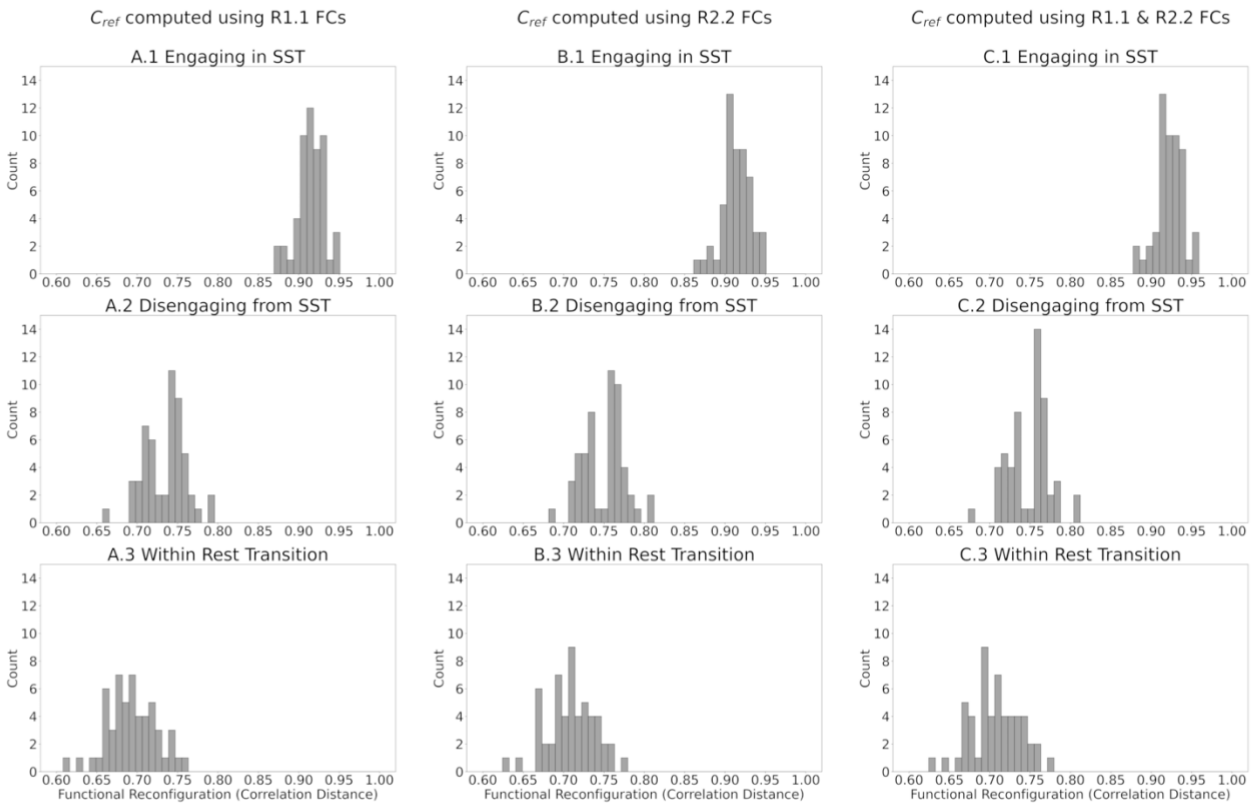

Figure S1. **(A1-A3)** Histograms of functional reconfiguration values of engaging to SST, disengaging from the SST, and within post-SST rest with C_{ref} as the Riemann mean of R1.1 FCs. **(B1-B3)** Histograms of functional reconfiguration values of engaging to SST, disengaging from the SST, and within post-SST rest with C_{ref} as the Riemann mean of R2.2 FCs. **(C1-C3)** Histograms of functional reconfiguration values of engaging to SST, disengaging from the SST, and within post-SST rest with C_{ref} as the Riemann mean of R1.1 and R2.2 FCs.

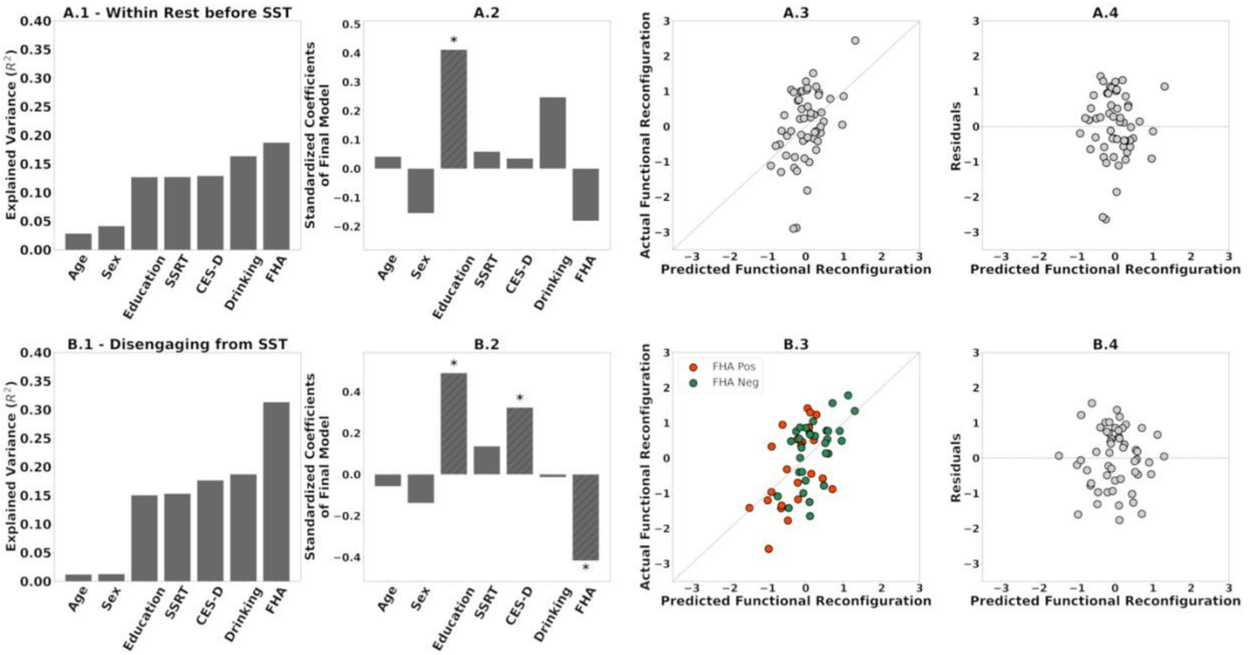

Figure S2. **(A.1, B.1)** Additive multilinear regression of AUD-risk variables (and adjustment covariates) on functional reconfiguration of within-rest before SST (R1.1 to R1.2) and disengaging from SST using the second rest segment after SST (SST to R2.2) with predictors sequentially introduced in the order depicted. See methods for variable definitions **(A.2, B.2)** Coefficients of the final multilinear regression model. Asterisks denote significant predictors ($p \leq 0.05$). **(A.3, B.3)** Scatter plots of predicted versus actual functional reconfiguration values for the final multilinear regression model in A1, B1, and C1 respectively. Colors in B.3 are based on FHA status. **(A.4, B.4)** Scatter plots of the predicted functional reconfiguration versus the standardized residuals of each participant for the final models in **A.1, B.1** respectively.

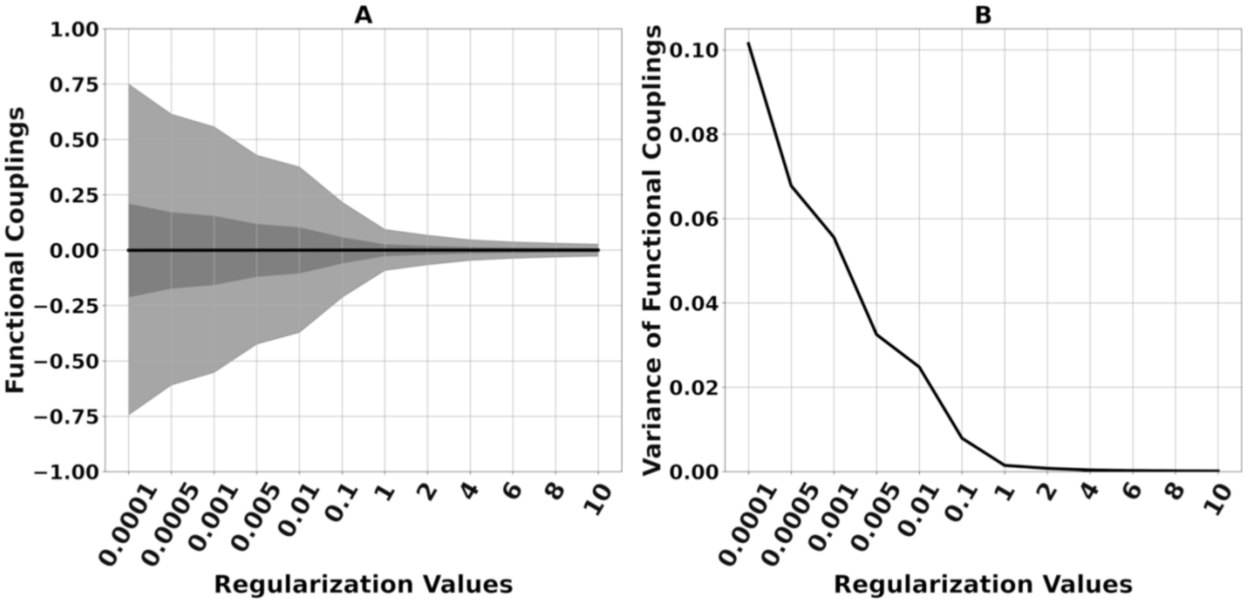

Figure S3. **(A)** Upper and lower bounds of functional couplings (elements of tangent-FCs) for each regularization value indicated by the shaded area spanning the 1st to 99th percentiles of functional couplings of all tangent FCs. The darker region shows the 25th to 75th percentile range. The black line indicates the mean. **(B)** Variance of functional couplings for each regularization value.
